# No Difference in Tolerance between Wheat and Spelt Bread in Patients with Suspected Non-Celiac Wheat Sensitivity

**DOI:** 10.3390/nu14142800

**Published:** 2022-07-07

**Authors:** Julia Zimmermann, Friedrich H. Longin, Anna Schweinlin, Maryam Basrai, Stephan C. Bischoff

**Affiliations:** 1 Institute of Nutritional Medicine, University of Hohenheim, Fruwirthstrasse 12, 70599 Stuttgart, Germany; julia.zimmermann@uni-hohenheim.de (J.Z.); anna.schweinlin@uni-hohenheim.de (A.S.); m.basrai@uni-hohenheim.de (M.B.); 2 State Plant Breeding Institute, University of Hohenheim, Fruwirthstrasse 21, 70599 Stuttgart, Germany; friedrich.longin@uni-hohenheim.de

**Keywords:** non-celiac wheat sensitivity, spelt, wheat, bread

## Abstract

Individuals with suspected non-celiac wheat sensitivity (NCWS) often report better tolerance of spelt (*Triticum aestivum* ssp. *spelta*) compared to wheat (*Triticum aestivum* ssp. *aestivum*) bakery products. This experience has neither been validated nor explained on a molecular level. Therefore, we performed blinded wheat and spelt bread challenge in this patient group. Twenty-four adults with a history of NCWS but suspected spelt tolerance were challenged in a single-blinded crossover design over six weeks with six different study breads each at 300 g per day for 4 days followed by a washout phase of 3 days. Study breads comprised spelt and wheat breads made either after a traditional (T) or a current (C) recipe, resulting in four bread types plus a gluten-free bread with 1.5% added oligosaccharides (+FODMAP) and a gluten-free bread with 5% added wheat gluten (+Gluten). The main outcome parameter was the Irritable Bowel Syndrome—Severity Scoring System, which was higher than self-estimated by the participants after spelt bread consumption (*p* = 0.002 for T; *p* = 0.028 for C) and lower for wheat bread (*p* = 0.052 for T; *p* = 0.007 for C), resulting in no difference between wheat and spelt bread tolerance. The +FODMAP bread was better tolerated than both T breads (*p* = 0.003 for spelt; *p* = 0.068 for wheat) and equally well tolerated as both C breads and +Gluten breads after normalization to the washout scores. Neither signs of inflammation nor markers for intestinal barrier integrity were influenced. Our data do not confirm, on an objective basis, the differences in expected symptoms resulting from wheat and spelt products, suggesting a strong nocebo effect for wheat and a placebo effect for spelt.

## 1. Introduction

Incidences of wheat-related disorders are increasing, with celiac disease (CD) and wheat allergy (WA) each affecting 1% of adults [1,2]; in addition, there has been an up to 13% increase in the prevalence of non-celiac wheat sensitivity (NCWS) in adults [3]. In contrast to CD and WA, the mechanisms of NCWS are unclear but an involvement of the immune system and an impaired intestinal barrier are likely [4,5]. Suspected triggers of symptoms in NCWS are gluten, fermentable oligo-, di-, monosaccharides and polyols (FODMAPs), and wheat α-amylase trypsin inhibitors (ATIs) [6,7,8].

Bread wheat (*Triticum aestivum* ssp. *aestivum*), hereafter referred to as “wheat”, and spelt wheat (*Triticum aestivum* ssp. *Spelta*), hereafter referred to as “spelt”, are hexaploid subspecies of the genus wheat (*T. aestivum*), and thus, are biologically closely related to each other. In a subgroup of NCWS patients, spelt bread is subjectively better tolerated than wheat bread [9,10]. Despite the great genetic similarity, spelt has a higher average crude protein content and a higher gliadin-to-glutenin ratio than wheat [11,12]. In internal proteome analyses, we could show that one third of the proteins differ between wheat and spelt flour [13], but the amount of suspected triggers of NCWS—such as gluten, FODMAPs, and ATIs in bread—seem to be influenced rather by the grain variety and growth conditions than by the choice between wheat and spelt [8,14,15], which could indicate further, as yet unknown triggers that are only found in wheat but not in spelt. Additionally, the manufacturing processes of bread such as milling, kneading, fermentation, heating and the addition of improver might affect the bread composition and the presence of possible triggers of NCWS in bread [16,17,18]. In particular, the long bread dough fermentation time is leading to a reduction in FODMAPs [14], which is why it is suspected to be tolerated better in some individuals.

This study aimed to investigate the hypothesis that spelt bread is better tolerated than wheat bread in individuals with suspected NCWS. For spelt bread, flour type 630 instead of 550 is used, and spelt doughs are often leavened longer than wheat doughs due to traditional production and to improve their rather poor technological performance [11], which may also account for the observed differences in spelt and wheat tolerance. Therefore, we also analyzed the effects of a “traditional” recipe with a long yeast fermentation time without bread improver and a “current” recipe characterized by short yeast fermentation time and the addition of bread improver. For this purpose, after blinded administration of different wheat and spelt breads, symptoms were assessed by the Irritable Bowel Syndrome—Severity Scoring System (IBS-SSS) questionnaire [19]. Extraintestinal symptoms and various blood and stool parameters were also analyzed. To reveal whether FODMAPs or gluten are potential triggers for symptoms in this group of patients, two additional breads, enriched with 1.5% oligofructose (+FODMAP) or 5% gluten (+Gluten), were also administered to the participants in a blinded manner.

## 2. Materials and Methods

### 2.1. Participants

Individuals between 18 and 70 years of age with subjective wheat intolerance and simultaneous spelt tolerance were eligible participants. This was confirmed by the IBS-SSS that was filled out by the participants based on the symptom experiences for wheat and spelt consumption before they were included in the trial. A difference of ≥50 points of IBS-SSS total score between anticipated symptoms after wheat compared to spelt consumption was considered as wheat intolerance and spelt tolerance. The study participants indicated an IBS-SSS score of >75 points in response to wheat bread consumption. Further, the patients had no evidence for CD, which was considered adequately excluded if the tissue transglutaminase (tTG) antibody test was negative while on a gluten-containing diet; for WA, which was excluded if serology showed negative wheat-specific immunoglobulin E (IgE) levels (g15, f4, and f98); and for other GI diseases (normal colonoscopy in patients with suspected GI disease, no drugs for GI diseases, and no history of GI disease). Indeed, all participants underwent a colonoscopy prior to the study based on medical indication because of sustained GI symptoms of an unclear origin. The exclusion criteria were a history of pregnancy and lactation as well as acute or chronic gastrointestinal (GI) comorbidities other than NCWS and the intake of specific drugs like immunosuppressive agents or antibiotics. The study took place from May 2020 to August 2021 at the University of Hohenheim in Stuttgart, Germany. Participants were recruited in cooperation with the Association of the Grain, Milling and Starch Industry. Recruitment was carried out by advertisements in trade journals and social media as well as by distributing flyers at bakeries, supermarkets, or mills. In addition, flyers and posters were sent to nutritionists and practitioners. There was no change in methods after trial commencement.

### 2.2. Study Breads

Study breads were produced by the “Akademie Deutsches Bäckerhandwerk” in Stuttgart, Germany (Table 1).

The purpose of the bread selection was to compare wheat and spelt bread, and more traditional versus current baking methods. Therefore, we used two different recipes (T, “traditional” and C, “current”) differing in fermentation time (1 h at 20 °C for C and 16 h at 4 °C for T) and the addition of bread improver to the breads based on the current recipe for the wheat and spelt breads. The bread improver consisted of guar gum, potato fiber, citric acid, mono- and diglycerides of fatty acids, calcium carbonate, rapeseed oil, ascorbic acid and enzymes (cellulase, alpha-amylase, xylanase, maltogenic amylase) and was provided by Schapfenmühle (Ulm, Germany). For detailed recipes, see Table S1. Two additional breads were administered to the participants that were based on a gluten-free (GF) mixture. To the +FODMAP bread, we added Oligofructose OraftiP95 (Beneo GmbH, Mannheim, Germany), and to the +Gluten bread, we added wheat gluten (Loryma GmbH, Zwingenberg, Germany). Both breads were kneaded and immediately baked without any time of fermentation. All breads were cut and immediately frozen after cooling down and stored at −20 °C until handing it out to the participants. The flours used (Stelzenmühle, Bad Wurzach, Germany) were mixtures of two varieties each (wheat varieties: “Reform” and “Patras”; spelt varieties: “Zollernspelz” and “Franckenkorn”) and from three cultivation sites, to exclude any influence by variety or growth conditions. The varieties were a representative selection that have been widely used in Germany in recent years. Regarding the taste and appearance, there were differences between the breads based on the GF mixture compared to the wheat and spelt breads. Within the GF breads and the wheat and spelt breads, however, blinding was possible (Figure S1). All breads were packed in the same neutral bags. We additionally analyzed the nutritional values, as well as the gluten and FODMAP contents of the breads (Table S2).

### 2.3. Study Design and Intervention

At baseline (visit 1), we systemically recorded the medical background of all participants, including, e.g., additional diseases, food intolerances, and wheat-related symptoms. Additionally, state of Irritable Bowel Syndrome (IBS) was assessed as defined by the Rome IV criteria at baseline. The IBS-SSS questionnaire was filled out at baseline (visit 1), after washout phases, and after a challenge with study breads (visits 2–7). Furthermore, the anticipated symptoms expected by the subjects after wheat and spelt consumption were assessed using the IBS-SSS scoring before the study started. All eligible participants identified wheat as a trigger for their GI symptoms in the past (without medical evidence), whilst tolerating spelt very well at the same time. So, all the participants were asked prior to the study to fill out the IBS-SSS questionnaire according to their experiences with wheat and spelt consumption. The results were IBS-SSS scores that were expected by the participants for wheat and spelt bread due to their subjective experiences. Additionally, a baseline measurement of blood and stool parameters was conducted as well as a 7-day food record before the study. Before the study’s initiation, each subject received nutritional education to ensure adherence to the gluten-free diet (GFD) and low-FODMAP diet (LFD) required for the study. GFD and LFD adherence was assessed during the study by trained dietitians and evaluated by the food record that was filled out by the participants daily.

**Figure 1:**
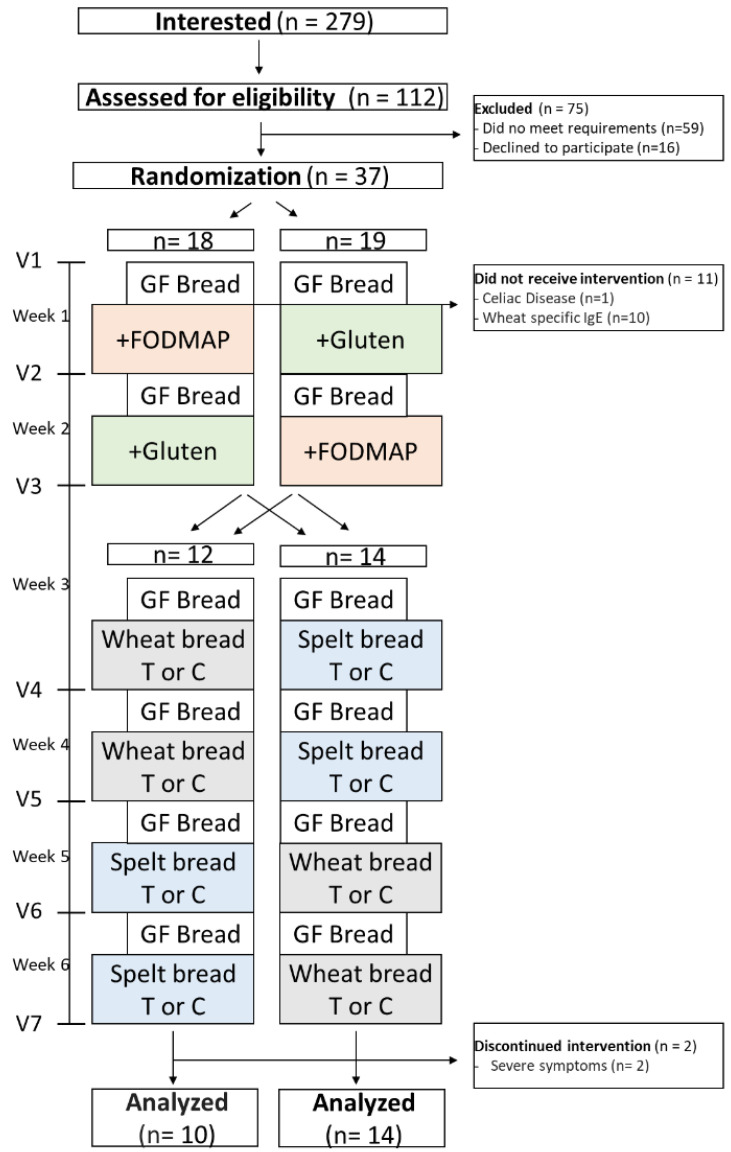
Study flow chart and design. GF, gluten-free; FODMAP, fermentable oligo-, di-, monosaccharides and polyols; T, traditional recipe; C, current recipe; V1–7, study visit 1–7. For further details, see text.

The study was conducted in a randomized single-blinded crossover design. Each study participant consumed the six study breads each on four consecutive days. A 3-day washout period with GF bread was conducted before and between the study bread challenges. The washout period could be extended on request by the participants until the symptoms, possibly induced by the previous study bread, were resolved. During the first two weeks, participants were randomly assigned 1:1 to the +Gluten or +FODMAP bread challenges (Figure 1).

In weeks 3 and 4, participants received either wheat breads or spelt breads, produced in a traditional or current manner, respectively. The order (first, traditional and second, current, or the other way round) was determined by chance. In weeks 5 and 6, participants who had wheat bread in weeks 3 and 4 received spelt bread, and participants who had spelt bread in week 3 and 4 received wheat bread. Again, the order (first, traditional and second, current, or the other way round) was determined by chance. Randomization was carried out using the online version of GraphPad Prism (La Jolla, CA, USA). The participants knew about the different bread types but were not informed about the logic behind the bread sequences. They were asked to consume 300 g of study bread per day (5–6 slices a day) corresponding to 15 g of gluten per day, as recommended for NCWS diagnosis [20]. The average daily gluten ingestion in humans is estimated to be between 13 and 30 g per day [8,21]. Since all other cereal products were forbidden during the study period, a consumption of 300 g of bread per day corresponds to the daily gluten consumption of the general population. The consumption of the same amount of GF bread in the washout phase should avoid a fundamental change in the nutritional behavior in the washout phase compared to the phases with study bread.

### 2.4. Questionnaire-Based Symptom Evaluation

Overall IBS-like symptoms, which were the primary outcome of the study, were measured by the validated IBS-SSS [19]. This questionnaire is structured as a five-subscore visual analogue scale (VAS) and evaluates the intensity of IBS-like symptoms regarding abdominal pain intensity and frequency, abdominal distension, bowel habits, and interference on life in general. Each of the five subscores generates a maximum score of 100 points, which gives a maximum IBS-SSS total score of 500 points. According to Francis et al. [19], a score between 75 and <175 was considered “mild IBS”, between 175 and 300 “moderate IBS”, and above 300 was defined as “severe IBS”. Further, they defined a significant change as a change of ≥50 points of the total score [19]. The subscore for abdominal pain frequency was designed for the evaluation of the last 10 days. For evaluating abdominal pain frequency in the washout phase with a maximum of 3 days and for the intervention phase with a maximum of 4 days, we multiplied the values for the washout phase by 3.3 and the values for the intervention phase by 2.5.

Secondary outcomes were extraintestinal symptoms measured by the brief Illness Perception Questionnaire (IPQ) [22], a nine-item scale designed to rapidly assess the cognitive and emotional representations of illness. The participants answered IBS-SSS and IPQ questionnaires at baseline, after every study bread (4 days), and after every washout phase (3 days). In addition, they answered the IBS-SSS before the study, regarding their expected complaints after consuming wheat or spelt bread. There were no changes in trial outcomes after the trial commenced.

### 2.5. Analysis of Fecal and Serum Markers of Intestinal Permeability and Inflammation

At baseline (visit 1) and after every study bread (visit 2–7), zonulin, lipase, ferritin, and anti-gliadin IgG antibodies were measured in the serum as well as calprotectin and lactoferrin in the feces. After the washout phases always placed prior to the study bread challenge phases and before fecal calprotectin and lactoferrin were measured. All measurements were performed in an accredited laboratory (Laborärzte Sindelfingen, Sindelfingen, Germany). Lipopolysaccharide-binding protein (LBP) was measured at baseline and after every study bread challenge using a commercial enzyme-linked immunosorbent assay kit (KR6813, Immundiagnostik AG, Bensheim, Germany) following the manufacturer’s protocols. For this purpose, 10 µL of blood plasma was used and processed as described [23].

### 2.6. Short-Chain Fatty Acids Analysis from Fecal Samples

At baseline and after weeks 4 and 6, the amount of short-chain fatty acids (SCFAs) in fecal samples was assessed as described elsewhere [24]. In short, raw fecal samples were homogenized, diluted 1:4 with distilled water, and 100 µL of 50% phosphoric acid (Carl Roth GmbH, Karlsruhe, Germany) was added. The supernatant was filtered with a syringe filter (WIC 79545, Wicom, Heppenheim, Germany) to an autosampler glass (WIC 42100 with crimp caps, Wicom) with Micro Inserts (No 548-00060, VWR International GmbH, Darmstadt, Germany). With a capillary gas chromatograph (Clarus 690, Perkin-Elmer, Waltham, MA, USA), 1 µL filtrate was analyzed. For data integration, the software TotalChrom Version 6.3.4 (Perkin-Elmer) was used. For fecal dry mass quantification, 200 mg of fecal sample was weighed and dried for 12 h at 103 °C. SCFA data were expressed in relation to dry mass and identified by comparing the retention times of the respective peaks in the sample and standard chromatograms.

### 2.7. Sample Size Calculation and Statistics

All data are shown as means ± SEM if not indicated otherwise. The required number of participants was calculated based on the IBS-SSS total score as the primary outcome by using data from a previous study, showing a difference of 79 score points and a standard deviation of 127 [25]. Assuming a similar difference in IBS-SSS total score between the different intervention phases with bread, we calculated the patient numbers needed in the present study using a statistical power (1−β) of 80% and a type I error rate α of 0.05, resulting in a group size of 23 participants using the software PS: Power and Sample Size Calculation version 3.1.6 (HyLown Consulting LLC., Atlanta, GA, USA). Loss to follow-up was kept to a minimum by regular visits every week; however, we expected a dropout rate of 35% during the follow-up, and therefore, included 37 patients. Testing for normal data distribution was performed using the Kolmogorov–Smirnov test. If normally distributed, paired data were compared by repeated measures one-way ANOVA with Tukey’s correction for multiple testing, otherwise by the Friedman matched-pair test with Dunn’s correction for multiple testing. A *p*-value of <0.05 was considered statistically significant. A *p*-value of <0.1 was considered as a trend. For statistical analysis and figure presentations, we used GraphPad Prism, version 9.2 (Graph Pad, La Jolla, CA, USA).

### 2.8. Compliance Testing

The participants were given 24 frozen slices of the study breads every week and were instructed to bring back leftover bread slices to the next study visit so that it was possible to calculate the consumed slices. In addition, we checked if they had complied with the gluten-free diet by gluten measurements in stool samples of 23 out of 24 participants (one did not deliver a stool sample at this time point) after the gluten-free washout phase following the +FODMAP bread phase (which means 10 days without gluten ingestion). For fecal gluten measurement, we used the IDK^®^ Gluten Fecal ELISA Kit (KT-5739, Immundiagnostik AG), which was performed following the manufacturer’s protocols. Results below 6 mg/g indicated a strictly gluten-free diet; results below 500 ng/g indicated a largely gluten-free diet (<50 mg Gluten/d) with small amounts of gluten due to contaminated oats or similar contaminants [26]. We considered the largely gluten-free diet to be sufficient for our study, since NCWS patients are not recommended to follow as strict of a gluten-free diet as celiac patients [20].

### 2.9. Ethics and Approvals

Written informed consent was obtained from all individuals involved in the present study. The study was conducted according to the Declaration of Helsinki, approved by the local Ethical Committee (Ethikkommission der Landesärztekammer Baden-Württemberg, Stuttgart, Germany), and registered on clinicaltrials.gov (accessed on 1 June 2022) (NCT04401956).

## 3. Results

### 3.1. Recruitment

Of the 112 participants assessed from May 2020 to August 2021, 59 participants did not meet the requirements (e.g., weekly attendance uncertain due to long travel distance, refused to consume wheat products due to uncertainty regarding whether they can tolerate them, only allowed medications, or not sure that spelt products are better tolerated than wheat products) and were excluded. Sixteen participants declined to participate. The remaining 37 participants were randomized into two groups, starting with consuming either +FODMAP bread or +Gluten bread, respectively (Figure 1).

Because of the situation caused by the COVID-19 pandemic in 2020, study visits were reduced to a minimum so that the diagnosis of CD and WA was carried out after the first study visit. Ten participants showed elevated levels of IgE antibodies against wheat and one participant had elevated levels of anti-tTG antibodies, leading to 11 participants which consumed GF breads but not the study breads. These participants were not included in the analysis. Of the remaining participants, two terminated the study prematurely because of unbearable symptoms (one after wheat bread C and one after spelt bread T). The remaining 24 participants completed the whole study and were included in the statistical analysis. During the challenges, all participants self-reported a strict adherence to a gluten-free diet, which was confirmed by fecal gluten ELISA, while the consumption of small amounts of gluten which could be based on contaminated oats or similar was accepted as a gluten-free diet (Figure S2).

### 3.2. Baseline Data

Baseline characteristics of the study population are shown in Table 2.

Most participants were female and were, on average, 42 years old, which is in line with the literature [10,27]. Their body mass index was within the normal range at 24.8 [28]. Two-thirds of the participants fulfilled Rome IV criteria for IBS and reported symptom relief on a wheat-free diet. More than one-third had already undergone gastroscopy without diagnosis and half of the participants suffered from further self-reported or diagnosed food allergies or intolerances. No one had a family member with a positive CD diagnosis. The time until the onset of symptoms was, on average, 5.6 h and they lasted on average for 19.2 h with a maximum of 72 h. The most frequent symptoms were bowel habit abnormalities (higher/lower stool frequency, stool consistency harder, or more liquid), bloating, systemic manifestations, and abdominal pain.

### 3.3. Primary Outcome

**Figure 2:**
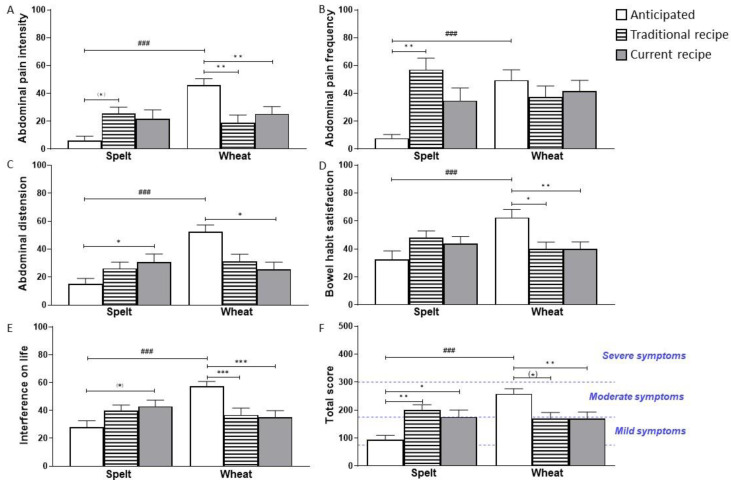
Results of Irritable Bowel Syndrome—Severity Scoring System (IBS-SSS) before (white) and after blinded consumption of traditional (patterned) and current (grey) wheat and spelt bread. The symptom scores for the 5 subscores (**A**–**E**) and IBS-SSS total score (**F**) as the sum of the 5 scores are shown. Statistics by *t*-test (only between anticipated scores of wheat and spelt) and Friedman/Dunn. ^(^*^)^ *p* < 0.1; * *p* < 0.05; ** *p* < 0.01; ^###^/*** *p* < 0.001.

The IBS-SSS results that were expected by the subjects before the study differed between wheat and spelt consumption regarding all five subscores and the total score (*p* < 0.001; Figure 2A–F).

While for spelt bread consumption, the participants expected only mild symptoms (>75 and ≤175 score points), they expected moderate symptoms (175–300 score points) for wheat bread consumption (Figure 2F). Comparing the expected and experienced IBS-SSS scores, we found lower scores after blinded wheat bread consumption. The interference on life was lower for both wheat breads (*p* < 0.001; Figure 2E) than expected, but abdominal pain intensity and bowel habit satisfaction also showed lower scores after blinded consumption (Figure 2A,D). Abdominal pain distension showed only lower scores for wheat bread C (Figure 2C); for abdominal pain frequency, there were no differences between the anticipated and the experienced strength in symptoms (Figure 2B). For the IBS-SSS total score, the differences between expectation and score after consumption were significant for produced wheat bread based on the current recipe (*p* = 0.007) and borderline significant for traditionally produced wheat bread by trend (*p* = 0.052; Figure 2F).

Considering the expected and experienced symptoms after spelt bread consumption, the opposite results were shown. The IBS-SSS total score was higher than expected for both spelt breads (*p* = 0.002 for T and *p* = 0.028 for C; Figure 2F). While the scores for abdominal pain intensity (*p* = 0.098; Figure 2A) and abdominal pain frequency (*p* = 0.004; Figure 2B) were higher than expected after spelt bread T, abdominal distension (*p* = 0.019; Figure 2C) and interference on life (*p* = 0.055) was increased by trend after spelt bread C.

Further evaluation of IBS-SSS questionnaires revealed a total score in a “mild IBS” range (>75 and <175 points) for the washout phase, the +FODMAP bread, and the two wheat breads, while the scores at baseline after the consumption of the +Gluten bread and after the two spelt breads reached a “moderate IBS” range (175–300 points) (Table 3). 

There were no significant differences between the six different breads, baseline, and the average washout phase after the blinded challenge test when using the Friedman test. However, when considering the significance criteria of Francis et al. [19], there was an increase for spelt bread T compared to +FODMAP bread and compared to the average score of the washout phases.

**Figure 3:**
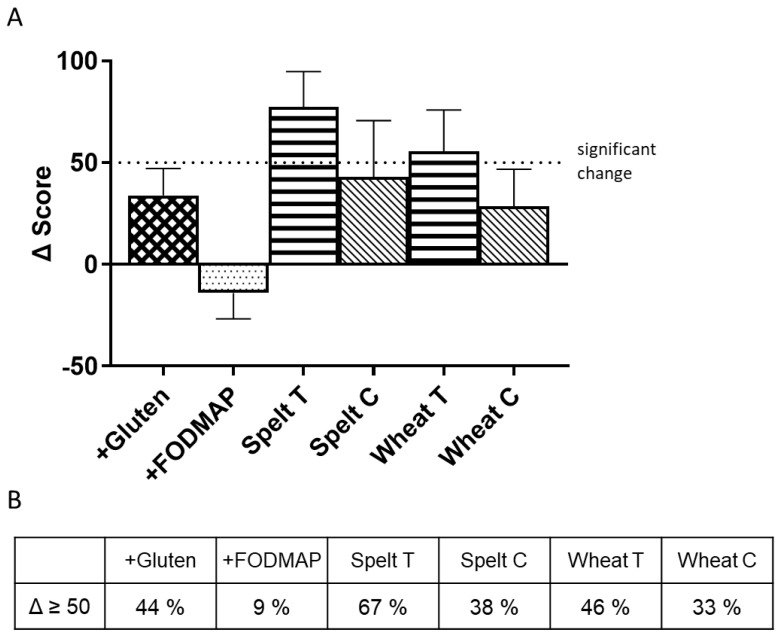
Total Score of Irritable Bowel Syndrome−Severity Scoring System (IBS-SSS) normalized to the previous washout phase for each individual (**A**) and percentage of participants with significantly elevated IBS-SSS total score (>50 points) (**B**). Abbreviations: FODMAP, fermentable oligo-, di-, monosaccharides and polyols; T, traditional recipe; C, current recipe. Statistics by Francis et al. (≥50 points) and Friedman/Dunn.

When we further normalized the IBS-SSS total score to the previous washout phase for each individual, we could show that symptom scores tended to improve after consumption of +FODMAP bread and significantly worsened after consumption of traditionally produced wheat and spelt bread (*p* = 0.068 for wheat and *p* = 0.003 for spelt; Figure 3A).

The differences between these two traditionally produced breads and the +FODMAP bread were statistically significant. Accordingly, the percentage of participants with elevated IBS-SSS total score compared to the previous washout phase was lowest after +FODMAP (9%) and highest after traditionally produced spelt bread (67%) (Figure 3B).

### 3.4. Secondary Outcomes

**Figure 4:**
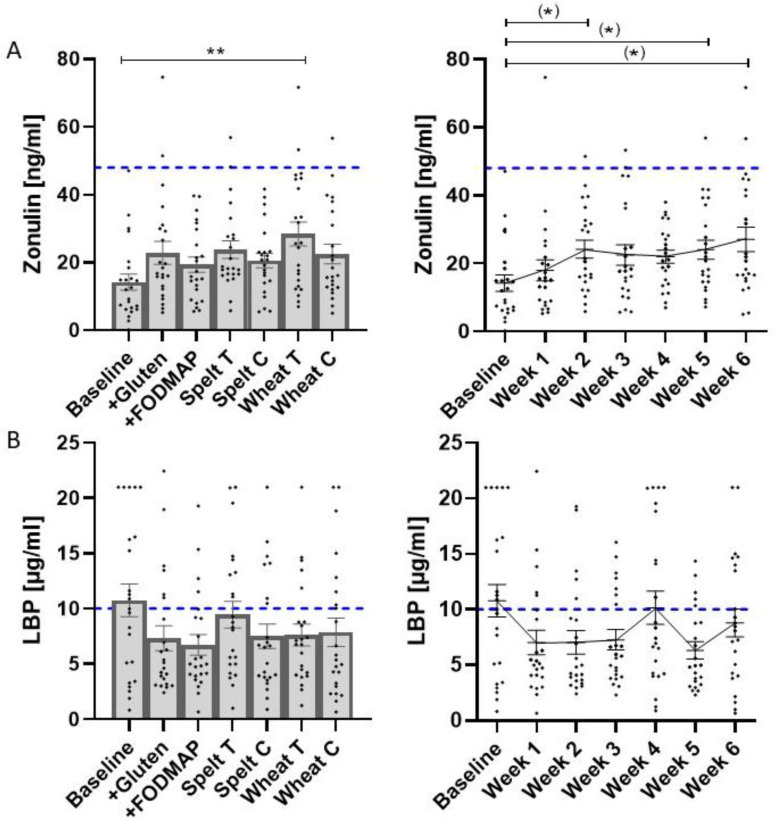
Parameters for gut permeability evaluation. The individual values for zonulin (**A**) and LBP (**B**) concentration, mean, and SEM after the different study breads (left) and over time (right) are shown. The reference values (<42 ng/mL for zonulin and <10 µg/mL for LBP) are shown by the dashed line. Abbreviations: LBP, lipopolysaccharide-binding protein; FODMAP, fermentable oligo-, di-, monosaccharides and polyols; T, traditional recipe; C, current recipe. Statistics by Friedman/Dunn. ^(^*^)^ *p* < 0.1; ** *p* < 0.01.

Regarding systemic manifestation of symptoms as evaluated by IPQ, and regarding laboratory values such as fecal calprotectin, fecal lactoferrin, serum ferritin and serum lipase, no differences occurred when comparing the effects of the different breads (Tables S3 and S4). None of the participants showed elevated levels of anti-gliadin IgG antibodies in the serum during the study (data not shown). The intestinal barrier marker, zonulin in serum, was increased after traditionally produced wheat bread exposure (*p* = 0.007) compared to baseline (Figure 4A, left), while a time-dependent effect could be shown by trend (Figure 4A, right).

Considering the reference value for serum zonulin (<42 ng/mL) for healthy individuals, as shown by the dashed line, we observed only a few individuals (0–8%) who had elevated zonulin levels. We further evaluated serum LBP concentration but there were no differences between the breads or between the study weeks (Figure 4B). Considering the reference value for serum LBP (<10 µg/mL) for healthy individuals, as shown by the dashed line, a high proportion of the individuals (17–48%) had elevated levels of LBP, with the highest proportion at baseline and the lowest after the +FODMAP bread.

Assessing the total amount of SCFA after two weeks spelt and two weeks wheat consumption, we detected an increase of isocaproic acid between baseline and week 6 (*p* = 0.013), as well as between baseline and two weeks of wheat consumption (*p* = 0.02) (Table 4).

## 4. Discussion

In the present study, we aimed to analyze the effects of different bread types in individuals with self-reported wheat sensitivity but spelt tolerance, in which wheat allergy and celiac disease were excluded.

Similar to the preclinical studies we performed earlier [29], wheat gluten consumption seemed to have no negative effects in the patients of the present study since the IBS-SSS results of the gluten-rich bread were lower than that of wheat and spelt breads. Accordingly, Molina-Infante and Carroccio [30] analyzed data from 10 double-blind, placebo-controlled, gluten-challenge trials, comprising 1312 adults of whom only 38 showed gluten-specific symptoms. In line with our results, they showed that 40% of the participants had a nocebo response. Additionally, Tovoli et al. [31] showed that a high proportion of NCWS patients, even if significantly attenuated by the GFD, still suffer from intestinal and extraintestinal symptoms years after the beginning of the GFD.

When consuming wheat bread in a blinded manner, our participants experienced fewer symptoms than expected. On the other hand, our participants were convinced they had a better tolerance of spelt bread, which was confirmed by a survey with 49 self-reported gluten-sensitive participants; of them, 35% reported a reduction in clinical signs when consuming spelt bread [10]. In our study, there were no differences regarding intestinal symptoms after blinded consumption between spelt and wheat bread, suggesting a placebo effect for spelt consumption in this patient group. The misperception of these patients is most likely due to the fact that it has often been claimed that ancient grains, including spelt, have better tolerability than modern wheat cultivars. This assumption is based on lay-press reports and studies such as that of Prandi et al., who showed that spelt contains fewer immunogenic or toxic peptide epitopes compared to durum wheat, wheat, einkorn, and emmer [32]. However, in these analyses, only flours of one variety each and had derived from only one place of cultivation were examined. Our group showed, for flours of 15 varieties each of wheat and spelt grown at three different locations, that the place of cultivation is the major determinant for about half of the proteome. However, for the proteins discussed as possible triggers of wheat allergy, baker’s asthma, and wheat sensitivity, the varieties of grains seem to be more influential than the type of grain (wheat or spelt), or the place of cultivation [13]. When analyzing the bread proteome, we could show that the difference between the wheat and spelt bread proteome, especially regarding immunogenic proteins, is quite small, while the difference to rye bread was more pronounced [16]. Additionally, regarding the FODMAP content of bread, the grain variety, and most importantly, the dough preparation, seem to be more important than the sub-species wheat or spelt [15].

Unexpectedly, no significant differences in GI symptoms occurred after the consumption of breads based on the traditional or current recipe. As our group already demonstrated for other breads [15], both the traditionally and currently produced wheat and spelt breads of our study had a rather low FODMAP content when considering the tolerance threshold reported by Varney et al. [33]. Only the oligosaccharide content of wheat bread C was above the threshold of 6 mg/g bread, while the +FODMAP bread contained amounts that exceeded the threshold for oligosaccharides by factor 8, and for excess fructose (3 mg/g), by factor 6 (Table S2). Interestingly, the +FODMAP bread induced the lowest symptom scores, which differed from those induced by traditional spelt bread containing particularly low amounts of FODMAP. This contradicts the dominant observation in the literature, which states that a FODMAP reduction results in a reduction in GI symptoms [7,10]. It should be noted, however, that in the present study, a low FODMAP baseline diet was followed during the whole study time, which might influence the results. Subsequently, we cannot exclude that the symptoms would have been more pronounced if a baseline diet with higher FODMAP content would have been installed.

As a secondary outcome, we considered validated markers for the intestinal barrier (serum LBP and zonulin) and inflammation (fecal calprotectin and lactoferrin, serum lipase and ferritin). While there were no differences between the breads regarding markers of inflammation, our research showed an increase in zonulin between baseline and following traditionally produced wheat bread consumption over time; this could be due to an accumulation effect. Zonulin is a human protein which is involved in the regulation of intercellular contacts (tight junctions) in the intestinal wall. An increased blood zonulin concentration is associated with an impaired intestinal barrier [34]. However, the increase in zonulin was rather marginal and remained largely within the normal range (<42 ng/mL), resulting in pathological zonulin levels in only 1–2 out of 24 participants over the course of the study. This means that we could not see the negative effects of any of the breads regarding the intestinal barrier in most study participants.

Dietary fibers pass to the large bowel to be fermented by the gut microbiota into metabolites, such as SCFAs and many others. Analyzing fecal SCFAs at baseline and after two weeks of study bread exposure of only wheat bread consumption increased a particular SCFA, isocaproic acid, indicating increased levels of *Clostridium difficile* in the gut [35]. In former analyses, it was shown that a fiber-rich diet lowers the prevalence of *Clostridium difficile* [36,37], leading to our hypothesis that the Low-FODMAP diet during the study promotes an increase of *Clostridium difficile* in the gut.

### Limitations

In the present trial, we could only include participants who agreed to consume wheat bread, while many interested individuals declined to participate because of the fear of GI symptoms being too heavy. This unintended factor of the selection of the study population could have influenced the results, since individuals who are highly sensitive to wheat might be excluded at higher rates. Compared to other trials in which the participants were challenged over 1 week [6,7,38], 4 days in our study was rather short, but like others [39,40], we were convinced that this time should be sufficient to produce symptoms and indeed we could induce the symptoms supporting our decision. A wash-in period longer than 3 days to reduce baseline symptoms prior to the challenges could be advantageous, as recommended by Bascunan et al. [41].

## 5. Conclusions

Our study shows that, in contrast to some expectations, there is no objective difference between wheat and spelt bread in terms of triggering GI symptoms in suspected NCWS patients. Moreover, traditionally produced breads were not better tolerated than bread that was based on a current recipe, leading to the conclusion that psychological effects may play an important role in the perception of symptoms in this patient group. Further, a positive influence on GI symptoms of a bread rich in oligosaccharides could be shown, warranting further investigations.

## Figures and Tables

**Table 1:** Composition of the gluten-free bread and the different study breads.

Bread	Description	Ingredients
Bread for washout-phase		
GF bread	Gluten-free bread based on a mixture (Dietary Food Solutions, Lana, Italy)	Corn starch, flaxseed flour, buckwheat flour, vegetable fibers (psyllium, apple, sugar beet, rice, pea), salt, rice sourdough, sugar, thickener (E464, pea protein, spices and alpha amylase)
Study breads		
+Gluten	GF bread supplemented with wheat gluten (Loryma GmbH, Zwingenberg, Germany)	Corn starch, flaxseed flour, buckwheat flour, vegetable fibers, salt, rice sourdough, sugar, thickener + 5% Wheat gluten
+FODMAP	GF bread supplemented with oligofructose (Beneo GmbH, Mannheim, Germany)	Corn starch, flaxseed flour, buckwheat flour, vegetable fibers, salt, rice sourdough, sugar, thickener + 1.5% Orafti^®^P95
Spelt T	Spelt bread produced according to a traditional recipe	Spelt flour (Type 630), water, yeast, salt; fermentation for 16 h at 4 °C
Spelt C	Spelt bread produced according to a current recipe with bread improver	Spelt flour (Type 630); water, yeast, bread improver (Schapfenmühle, Ulm, Germany), salt; fermentation for 1 h at 20 °C
Wheat T	Wheat bread produced according to a traditional recipe	Wheat flour (Type 550); water, yeast, salt; fermentation for 16 h at 4 °C
Wheat C	Wheat bread produced according to a current recipe with bread improver	Wheat flour (Type 550); water, yeast, bread improver (Schapfenmühle, Ulm, Germany), salt; fermentation for 1 h at 20 °C

Abbreviations: GF, gluten free; FODMAP, fermentable oligo-, di-, monosaccharides and polyols; T, traditional recipe; C, current recipe.

**Table 2:** Baseline characteristics (*n* = 24).

Parameter	Proportion of Study Participants
Female/male	23/1
Age (y), SD	42 (±14.1)
Mean body mass index (kg/m^2^), SD	24.8 (±6.8)
IBS by Rome IV criteria (%)	67
Previous gastroscopy (%)	38
Other food allergy/intolerance (%)	54
Family member with celiac disease (%)	0
Time until onset of symptoms (h)	0–24 (Ø 5.6)
Duration of symptoms (h)	0.5–72 (Ø 19.2)
Main symptoms after wheat consumption	
Abdominal pain (%)	38
Bloating (%)	71
Bowel habit abnormalities (%)	75
Systemic manifestations (%)	71

Abbreviations: IBS, Irritable Bowel Syndrome.

**Table 3:** Results of Irritable Bowel Syndrome—Severity Scoring System (IBS-SSS).

	Baseline	+Gluten	+FODMAP	Spelt Bread T	Spelt Bread C	Wheat Bread T	Wheat Bread C	Wash Out Ø	*p*-Value
Abdominal pain intensity (max. 100)	18.8 ± 4.0	22.2 ± 5.9	14.8 ± 4.5	25.7 ± 3.9	21.9 ± 5.5	19.1 ± 5.0	25.4 ± 4.8	15.1 ± 2.3	n.s.
Abdominal pain frequency (max. 100)	29.1 ± 5.3	42.7 ± 8.1	37.3 ± 8.3	57.1 ± 8.3	34.8 ± 9.1	37.3 ± 8.1	41.8 ± 7.5	34.1 ± 4.6	n.s.
Abdominal distension (max. 100)	32.9 ± 4.8	34.1 ± 5.9	25.0 ± 5.6	27.2 ± 4.4	30.7 ± 5.5	31.5 ± 4.8	27.1 ± 5.3	22.3 ± 2.4	n.s.
Dissatisfaction of bowel habit (max. 100)	46.6 ± 4.7	40.0 ± 5.1	38.3 ± 5.1	48.5 ± 4.7	44.2 ± 4.8	40.9 ± 4.6	40.4 ± 4.7	36.0 ± 2.7	n.s.
Interference on life in general (max. 100)	46.5 ± 3.9	39.1 ± 4.1	35.9 ± 5.1	39.7 ± 3.9	43.5 ± 4.4	37.7 ± 4.7	35.8 ± 4.6	31.3 ± 3.0	n.s.
IBS-SSS total score (max. 500)	176.3 ± 18.1	177.2 ± 24.8	149.4 ± 23.5	198.7 ± 18.8	177.7 ± 25.7	165.0 ± 23.1	167.3 ± 23.4	135.7 ± 17.8	n.s.

Means ± SEM are shown. Abbreviations: FODMAP, fermentable oligo-, di-, monosaccharides and polyols; T, traditional recipe; C, current recipe; n.s., not significant. Statistics by Friedman/Dunn.

**Table 4:** Concentration of short-chain fatty acids (SCFA) per dry mass in fecal samples at baseline, after week 4 and week 6 as well as after consumption of two weeks spelt bread (spelt) and after two weeks wheat bread (wheat).

Fecal SCFA (*n* = 24)							
µmol/g DM	Baseline	Week 4	Week 6	*p*-Value	Spelt	Wheat	*p*-Value
Total SCFA	476.7 ± 362.5	462.5 ± 327.8	512.9 ± 442.1	n.s.	486.8 ± 38.2	488.6 ± 334.8	n.s.
Acetic acid	314.0 ± 244.4	285.6 ± 95.6	326.0 ± 290.6	n.s.	306.0 ± 284.5	305.9 ± 206.5	n.s.
Propionic acid	72.0 ± 56.3	80.5 ± 67.0	86.9 ± 89.0	n.s.	82.4 ± 88.0	85.0 ± 85.0	n.s.
Iso-butyric acid	7.6 ± 3.1	9.7 ± 7.5	8.7 ± 4.9	n.s.	8.6 ± 4.1	9.82 ± 8.0	n.s.
Butyric acid	60.8 ± 61.0	59.3 ± 49.6	64.0 ± 55.0	n.s.	63.5 ± 61.1	59.8 ± 41.8	n.s.
Iso-valeric acid	10.2 ± 4.6	13.2 ± 10.7	11.1 ± 6.1	n.s.	11.3 ± 5.2	12.9 ± 11.2	n.s.
Valeric acid	8.3 ± 3.6	9.6 ± 9.1	10.3 ± 10.8	n.s.	10.1 ± 10.8	9.7 ± 9.2	n.s.
Iso-Caproic acid	0.6 ± 0.8 ^(a)/(c)^	0.7 ± 0.6 ^(a)^	1.2 ± 1.2 ^(b)^	0.013 *	0.8 ± 2.2 ^(c)^	1.2 ± 1.2 ^(d)^	0.020 *
Hexanoic acid	2.8 ± 2.4	3.1 ± 4.5	3.8 ± 5.5	n.s.	3.3 ± 5.6	3.6 ± 4.4	n.s.
Heptanoic acid	0.5 ± 0.4	0.7 ± 0.7	0.6 ± 0.8	n.s.	0.7 ± 0.8	0.6 ± 0.7	n.s.

Statistics by Friedman/Dunn. Different letters (a + b) indicate statistical difference based on post hoc analyses (*p* < 0.05) for baseline, week 4, and week 6; the letters c + d indicate statistical difference to baseline, spelt and wheat consumption. * *p* < 0.05. Abbreviations: DM, dry mass; n.s., not significant.

## Data Availability

The data presented in this study are available on justified request to the corresponding author.

## Supplementary Materials

The following supporting information can be downloaded at: https://www.mdpi.com/article/10.3390/nu14142800/s1, Table S1: Composition of the wheat and spelt breads; Table S2: Content of nutrients, gluten and FODMAPs; Table S3: Systemic symptoms assessed by an Illness Perception Questionnaire (IPQ); Table S4: Fecal levels of calprotectin and lactoferrin as well as concentrations of ferritin and lipase in the serum of the participants; Figure S1: Gluten-free bread and study breads; Figure S2: Fecal levels of 33-mer peptides.
