# Supplementary material for: No Difference in Tolerance between Wheat and Spelt Bread in Patients with Suspected Non-Celiac Wheat Sensitivity
Source: Nutrients. 2022 Jul 7;14(14):2800. doi: 10.3390/nu14142800 (PMC9319925; doi:10.3390/nu14142800)
Supplement: Supplementary file 1 [file nutrients-14-02800-s001.zip › nutrients-1776379-supplementary.pdf]

Supplements

Table S1. Composition of the wheat and spelt breads

Name	Grain	Flour type	Dough	Improver [g]	Flour [g]	Yeast [g]	Salt [g]	Water [g]	Fermentation time
Spelt T	Spelt wheat	630	Yeast dough	-	5000	125	100	3500	16 h at 4°C
Spelt C	Spelt wheat	630	Yeast dough	125	3500	125	100	3500	1 h at 20°C
Wheat T	Bread wheat	550	Yeast dough	-	5000	125	100	3500	16 h at 4°C
Wheat C	Bread wheat	550	Yeast dough	125	3500	125	100	3500	1 h at 20°C

Abbreviations: T, traditional recipe; C, current recipe.

Table S2. Content of nutrients, gluten and FODMAPs

	Gluten g/100g	Polyols g/100g	Excess Fructose g/100g	Oligosaccharides g/100g	Energy KJ/100g	Protein g/100g	Fat g/100g	Carbohydrate g/100g
GF Bread	<0.05	0.52	0.08	0.62	841.3	4.1	1.3	38.91
GF Bread + Gluten	4.8	0.65	0.09	0.69	979.7	6.5	1.6	41.55
GF Bread + FODMAP	<0.05	0.30	1.81	5.15	922.5	4.1	1.3	43.21
Spelt bread T	5.3	0.36	0.00	0.39	1107.1	7.5	1.3	48.18
Spelt bread C	5.7	0.17	0.12	0.56	1065.3	7.3	1.1	48.41
Wheat bread T	5.6	0.32	0.06	0.40	1029.1	7.1	0.9	47.06
Wheat bread C	5.8	0.27	0.18	1.14	1060.1	7.3	1.1	48.42

For abbreviations, see Figure S2.

Table S3. Systemic symptoms are assessed by Illness Perception Questionnaire (IPQ). Shown are the numbers of affected individuals who suffered from the corresponding symptoms after each bread.

	Baseline n=24	+Gluten n=24	+FODMAP n=24	Spelt bread T n=24	Spelt bread C n=24	Wheat bread T n=24	Wheat bread C n=24	Wash out Ø n=24
Pain	18	10	9	14	10	10	12	10
Sore Throat	3	1	0	1	0	0	0	0
Nausea	13	5	3	4	3	3	5	3
Breathlessness	3	2	1	0	2	2	1	0
Weight Loss	3	0	0	1	2	3	2	0
Fatigue	8	6	4	3	5	5	4	3
Stiff Joints	5	2	0	2	3	5	2	0
Sore Eyes	2	1	1	1	2	1	0	0
Wheeziness	3	1	1	0	2	0	2	0
Headaches	10	3	6	3	6	3	6	3
Upset Stomach	18	14	11	16	12	11	15	10
Sleep Difficulties	6	3	2	2	2	5	6	1
Dizziness	5	1	2	2	3	1	3	0
Loss of Strength	9	5	3	3	4	3	2	2

For abbreviations, see Figure S2.

Table S4. Fecal levels of calprotectin and lactoferrin as well as concentrations of ferritin and lipase in the serum of the participants.

	Baseline	+Gluten	+FODMAP	Spelt T	Spelt C	Wheat T	Wheat C	P-Value
Calprotectin [mg/kg]	20.4±17.5	30.4±46.8	16.6±5.35	20.6±14.1	32.0±57.5	29.5±44.7	19.9±12.2	0.371
Lactoferrin [mg/l]	2.0±0.2	2.0±0.0	2.0±0.0	2.3±1.3	2.0±0.2	2.2±1.0	2.1±0.5	0.575
Lipase [U/l]	33.8±8.2	30.7±9.2	32.5±14.6	32.5±11.0	34.0±9.5	32.8±9.3	31.1±10.5	0.571
Ferritin [μg/l]	103.9±136.7	102.5±131.5	114.3±155.3	94.9±115.1	92.7±121.4	88.8±107.4	89.5±105.8	0.081

Statistics by ordinary one-way ANOVA/Tukey. For abbreviations, see Figure S2.

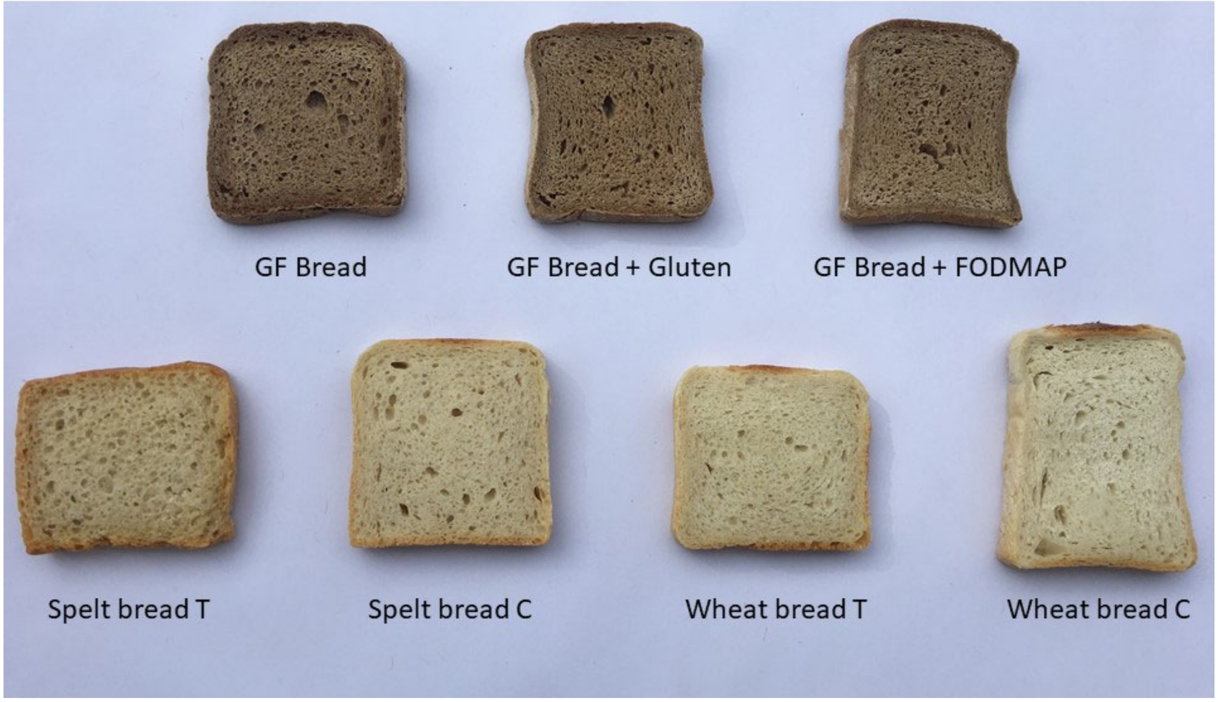

Figure S1. Gluten-free bread and study breads. Abbreviations: GF, gluten-free; FODMAP, fermentable oligo-, di-, monosaccharides and polyols; T, traditional manufacturing; C, current manufacturing.

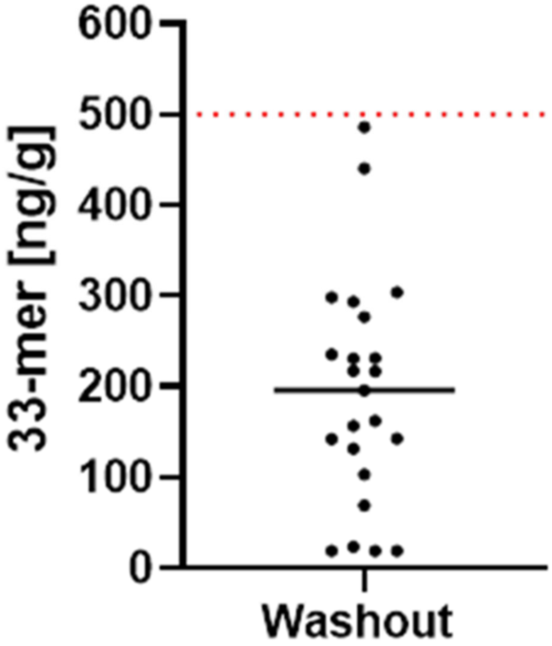

Figure S2. Fecal levels of 33-mer peptides. Measurement was performed after the washout phase after the +FODMAP bread (10 days of gluten-free diet).
